# Magnetic Resonance Imaging Has Utility in Clinical Decision-Making for Children and Adolescents With Symptomatic Subfibular Ossicles

**DOI:** 10.7759/cureus.67498

**Published:** 2024-08-22

**Authors:** James G Gamble, Kevin G Shea, Steve L Frick

**Affiliations:** 1 Department of Orthopaedic Surgery, Center for Academic Medicine, Pediatric Orthopaedic Surgery, Stanford, USA

**Keywords:** fibula chip fractures, ankle instability, pediatric ankle sprains, ankle mri, subfibular ossicles

## Abstract

Abstract: Treatment of children with chronic ankle pain, lateral instability, and the presence of a subfibular ossicle (SO) can be challenging. When will these patients respond to nonoperative management, and when will they require surgery? The purpose of this study was to investigate the utility of magnetic resonance imaging (MRI) in clinical decision-making for patients with symptomatic SOs.

Methods: We performed a retrospective study of pediatric patients with lateral ankle pain, recurrent sprains, and radiographs showing SOs who had undergone an MRI as part of their diagnostic work-up. We identified 22 patients from the senior author’s registry of pediatric patients with lateral ankle injuries. Eleven were girls, and 11 were boys, ages ranging from five to 19 years. Eleven involved the left ankle; 11 involved the right. Positive MRIs showed a high-intensity signal between the SO and the distal fibular epiphysis; negative MRIs had a low-intensity signal. The main outcome measure was operative versus non-operative treatment.

Results: Sixteen of the 22 patients had positive MRIs, and six had negative MRIs. Twelve of the 16 patients with positive MRIs had undergone an operation. All six patients with negative MRIs responded to nonoperative management.

Conclusions: MRI has utility in clinical decision-making for symptomatic patients with SOs. Patients with negative MRI sequences responded to nonoperative management. Most patients with positive MRI sequences will require surgery to alleviate their symptoms and return to full activities.

Level of evidence: IV

## Introduction

Children and adolescents commonly sustain inversion ankle injuries that cause sprains or lateral malleolus fractures [1,2,3,4]. Approximately 1% of pediatric patients sustaining lateral malleolus fractures will go on to form post-traumatic subfibular ossicles (SOs), especially after sustaining chip-type avulsion fractures [5,6]. SOs are small, well-marginated ossific densities located near the tip of the fibula and associated with chronic lateral ankle pain [6,7,8,9,10,11,12,13]. 

Reports in the literature are inconclusive as to which patients will respond to nonoperative management and which will need an operation to alleviate their symptoms [5,8,9,11,13,14,15]. The purpose of this report is to present our results concerning the utility of magnetic resonance imaging (MRI) in clinical decision-making for pediatric patients with lateral ankle pain, ankle instability, and radiographs showing SOs. Our hypothesis was that MRI is valuable in guiding clinical decision-making as to which patients will most likely need an operation to resolve their symptoms and which patients will respond to nonoperative management.

## Materials and methods

This study was conducted at Lucile Packard Children's Hospital Stanford, California. After receiving institutional review board approval from Stanford University School of Medicine (IRB-51263), we performed a retrospective study of children registered by the senior author (JGG) in an ankle injury database who were seen from January 2014 to June 2019. Patients were eligible for the study if (1) they had a history of lateral ankle pain and recurrent sprains, (2) they had radiographic evidence of an SO, and (3) they had undergone an MRI as part of their clinical evaluation.

We identified 22 eligible patients, 11 girls and 11 boys, ranging in age from five to 19 years. Eleven patients had SOs involving the left ankle, and 11 had SOs involving the right ankle. The exact date and mechanism of the initial injury were often unclear since most of the patients came to us later after developing chronic symptoms. Initially, we recommended that all the patients participate in ankle rehabilitation exercises under the supervision of a registered physical therapist. An MRI was ordered if the patient failed to improve after a minimum of six weeks of physical therapy.

**Figure 1:**
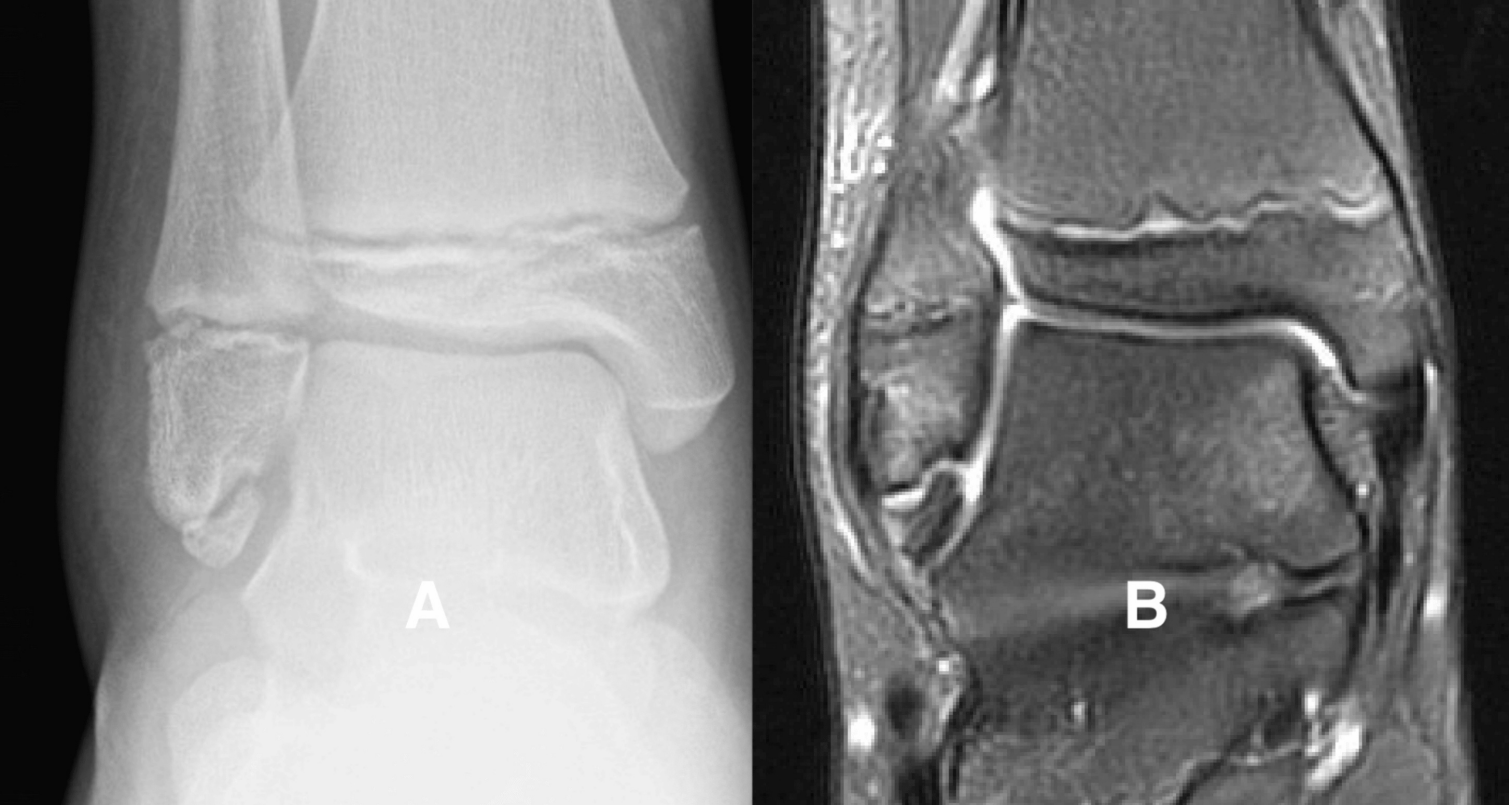
High-intensity fluid sensitive signal between the subfibular ossicle (SO) and the tip of the fibula. Figure 1A is a radiograph showing a well-formed SO present at the tip of the fibula. Figure 1B is a T2-weighted MRI sequence showing a high-intensity signal between the SO and the tip of the fibula.

All MRI studies were obtained on a 3 Tesla scanner using our institutional MR protocol for ankle imaging. The images we chose to evaluate for this study were fluid-sensitive sequences with a time to repeat of 3000 milliseconds and a time to echo of 80 milliseconds. We considered the MRI to be positive if these fluid-sensitive sequences showed a high-intensity signal between the SO and the tip of the fibula (Figure 1).

**Figure 2:**
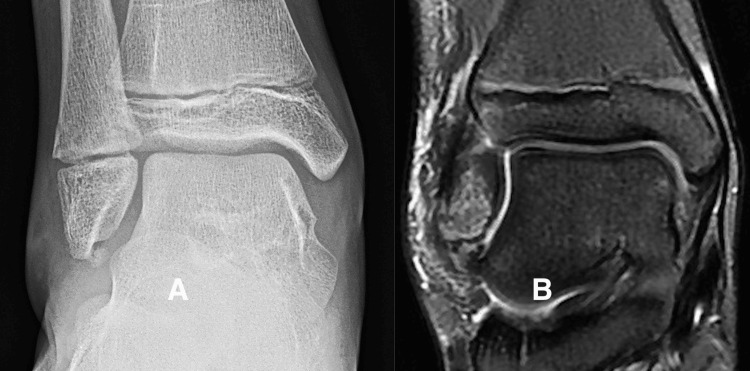
Low-intensity signal between the subfibular ossicle (SO) and tip of the fibula Figure 2A is a radiograph showing an SO at the tip of the fibula. Figure 2B is a coronal T2-weighted MRI sequence showing a low-intensity signal in the gap between the SO and the tip of the fibula. The child had recently sustained an inversion ankle sprain, and the MRI sequences showed soft tissue and bone edema related to the recent injury but no increased signal intensity between the SO and the fibula.

We considered the MRI to be negative if the sequences showed a low-intensity signal between the SO and the tip of the fibula (Figure 2).

We divided the 22 eligible patients into two groups. Group 1 had positive MRI sequences, and Group 2 had negative MRI sequences. We measured the maximum width and length of the SO using the embedded software in our institutional picture archiving and communication system. We evaluated coronal, axial, and sagittal MRI sequences to determine if the anterior talofibular ligament (ATFL) is attached to the SO.

Since the date of the injury was unclear, we recorded the length of follow-up as the date from obtaining the MRI to the date of the latest evaluation. We defined return to full activity as release to unrestricted participation in school physical education or return to unrestricted recreational sports participation.

We utilized Fisher’s exact tests and two-sample t-tests to compare the two groups. Statistical significance was set to 0.05.

## Results

Table 1 lists the demographic data for the 16 children in Group 1 who had positive MRI sequences, and Table 2 lists the data for the 6 children in Group 2 who had negative MRI sequences.

**Table 1:** Demographic data and outcomes for the 16 children with positive MRI sequences. MRI: magnetic resonance imaging, ATFL: anterior talofibular ligament

Case	Age (months)	Gender	Side	Sport	Ossicle width (mm)	Ossicle length (mm)	MRI result	ATFL attached	Treatment	follow-up (months)	Release to full activity
1	75	Female	Left	Soccer	6.5	5.5	Positive	Yes	Operation	20	Yes
2	80	Female	Left	Soccer	11	12	Positive	Yes	Operation	19	Yes
3	97	Male	Right	Soccer	6.8	8	Positive	Yes	Observation	34	Yes
4	119	Male	Left	Soccer	7	5	Positive	Yes	Operation	26	Yes
5	120	Male	Left	Football	5.9	6.4	Positive	Yes	Operation	12	Yes
6	131	Male	Right	Running	4.9	3.5	Positive	No	Observation	15	Yes
7	134	Male	Left	Soccer	4.1	9.5	Positive	Yes	Operation	33	Yes
8	157	Female	Right	Field hockey	7.7	10.1	Positive	Yes	Operation	13	Yes
9	162	Female	Left	Soccer	3.8	7.9	Positive	Yes	Operation	18	Yes
10	164	Female	Left	Soccer	3.9	8.7	Positive	Yes	Operation	24	Yes
11	170	Male	Right	Soccer	10	13.5	Positive	No	Observation	20	Yes
12	180	Female	Left	Soccer	9.8	3.7	Positive	Yes	Operation	12	Yes
13	184	Female	Right	Golf	7.3	5.6	Positive	Yes	Observation	16	Yes
14	200	Female	Right	Running	9.5	8.4	Positive	Yes	Operation	15	Yes
15	204	Female	Left	Basketball	6.5	11.7	Positive	Yes	Operation	24	Yes
16	230	Male	Left	Soccer	8.3	10.7	Positive	Yes	Operation	25	Yes

**Table 2:** Demographic data and outcomes for the six children with negative MRI sequences

Case	Age (months)	Gender	Side	Sport	Ossicle width (mm)	Ossicle length (mm)	MRI result	ATFL attached	Treatment	Follow-up (months)	Release to full activity
1	61	Female	Right	Basketball	4	7.5	Negative	No	Observation	16	Yes
2	108	Male	Right	Soccer	3	8	Negative	No	Observation	38	Yes
3	138	Male	Right	Soccer	5.6	6	Negative	No	Observation	29	Yes
4	152	Male	Right	Basketball	5	4.8	Negative	No	Observation	26	Yes
5	170	Female	Left	Soccer	4.4	7.2	Negative	Yes	Observation	13	Yes
6	170	Male	Right	Soccer	2.0	34.1	Negative	No	Observation	19	Yes

Table 1 lists the demographic data for the 16 children in Group 1 who had positive MRI sequences, and Table 2 lists the data for the 6 children in Group 2 who had negative MRI sequences. We found no significant differences between the groups for ages at presentation nor the lengths of follow-up after obtaining the MRI. The most frequent activity at the time of injury for the patients in both groups was soccer (14/22) followed by basketball (3/22). 

Size of the SOs

The size and the shape of the SOs were variable in both groups, but the mean length and width of Group 1 ossicles were greater than Group 2 using the two-sample t-test. Group 1 ossicles' mean length was 6.9 mm with a standard deviation (SD) of 2.2 mm, and that of Group 2 was 4.0 mm with SD of 1.3 mm, with p-value = 0.002. The mean width of Group 1 ossicles was 8.4 mm SD 2.9 mm, and that of Group 2 ossicles was 6.3 mm with SD of 1.6 mm, with p-value = 0. 041.

ATFL attachment

MRI sequences showed that 14 of 16 children in Group 1 had direct attachment of the ATFLs to the SOs, but only one of the Group 2 children had images showing direct attachment with the other 5 showing a normal attachment of the ATFL to the fibula. 

Clinical decision-making and outcomes

Both Group 1 and Group 2 children were offered the option of non-operative treatment after reviewing the MRI results. Four families in Group 1 and all in Group 2 chose nonoperative treatment. Twelve families in Group 1 (75%) chose surgical treatment. Direct observation at the time of surgery confirmed the complete attachment of the ATFL to the SO in all cases. Eleven of the 12 children undergoing surgery had excision of the ossicle, and one (Case 16) had internal fixation based on the large size of the ossicle. All those who underwent surgery had a Broström Gould-type augmentation of the ATFL repair. At the latest follow-up, all the children of both groups had been released to return to full activity and had remained asymptomatic.

Case presentations

The following three cases illustrate the radiographic and clinical features of Group 1 children with MRI-positive sequences.

Case 1: Group 1, Case 5

**Figure 3:**
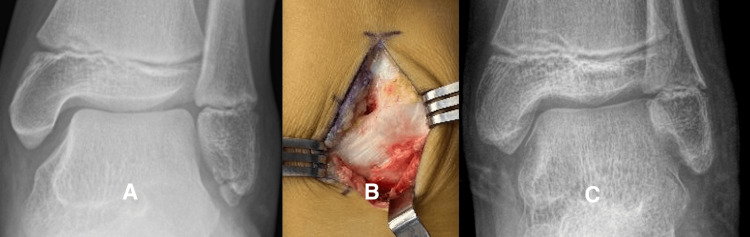
Representative case: patient group 1, case 5 Figure 3A is a radiograph showing the subfibular ossicle of patient Group 1, case 5. The MRI (not shown) was positive. Figure 3B shows an image of the ATFL attaching directly to the SO, and the motion was present between the SO and the fibula when the ankle was inverted.  Figure 3C is a radiograph of the latest evaluation after the patient had undergone surgery and had been released to return to full activity.

This 10-year-old boy had lateral ankle pain and recurrent sprains, and he did not improve after physical therapy. He and his parents were frustrated by his inability to return to full activity. After surgery, he was able to return to full sports participation (Figure 3).

Case 2: Group 1, Case 14

**Figure 4:**
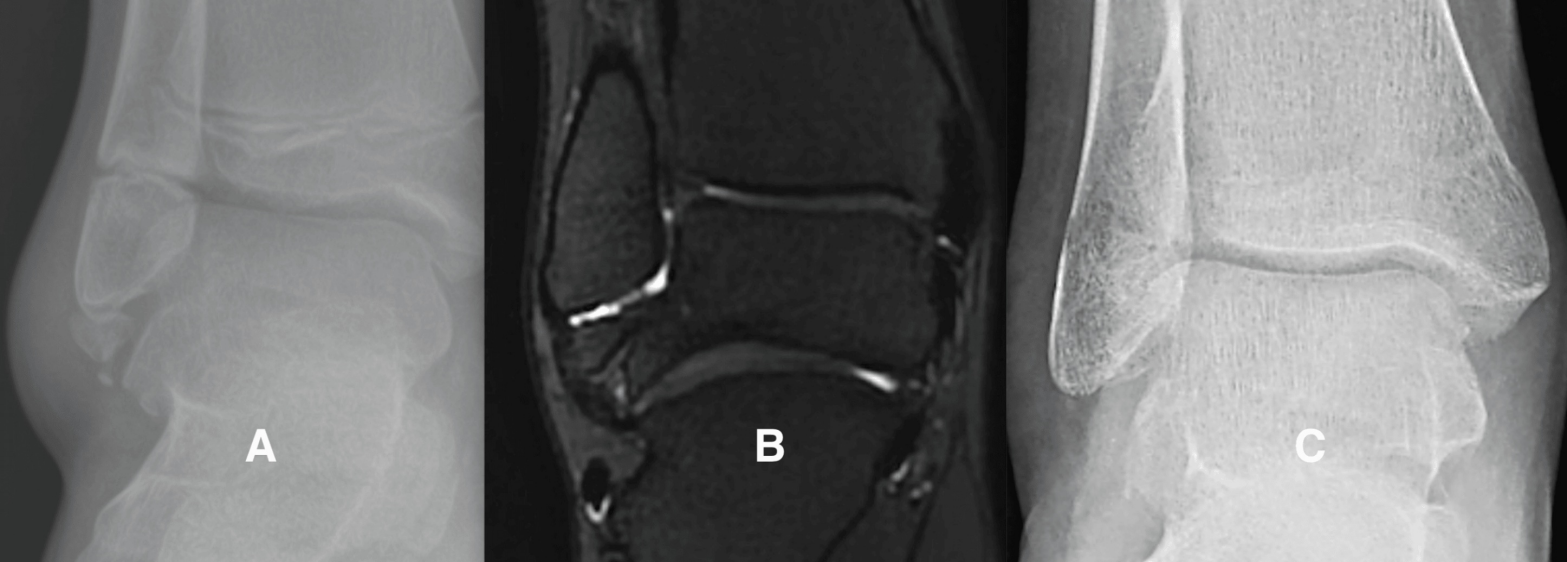
Representative case: patient group 1, case 14 Figure 4A is a radiograph of the right ankle of patient Group 1, case 14, after a recent ankle sprain showing soft tissue swelling and a well-formed SO with dystrophic calcification. Figure 4B is a fluid-sensitive MRI sequence with a high-intensity signal between the SO and the tip of the fibula.  Figure 4C is a radiograph at the latest follow-up after she had been released to full activity.

This 16-year-eight-month-old girl had sustained multiple right ankle sprains and complained of pain and lateral ankle instability. She wore an ankle brace, which did not prevent recurrent inversion sprains. This case illustrates a large SO with dystrophic calcification within the ATFL that had to be excised (Figure 4).

Case 3: Group 1, Case 16

**Figure 5:**
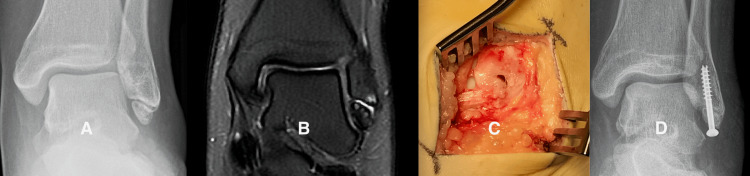
Representative case: patient group 1, case 16 Figure 5A is a radiograph of the left ankle of patient Group 1, case 16, showing a well-corticated, large subfibular ossicle (SO). Figure 5B is a fluid-sensitive MRI sequence showing a high-intensity signal in the gap between the ossicle and the tip of the fibula. Figure 5C shows the ATFL attaching directly to the SO with a gap between the SO and the tip of the fibula. Figure 5D is a postoperative radiograph showing the union of the SO at the time the patient was released to full activity.

This 17-year-old girl had sustained multiple lateral ankle sprains and had such severe chronic ankle pain that she was receiving care from our pain clinic. At surgery, we found the ATFL attached to a large SO with a gap between the SO and the tip of the fibula. We used internal fixation in this case due to the large size of the fragment and the presence of articular cartilage on the medial border (Figure 5).

## Discussion

SOs are irregularly shaped, ossific densities located at the tip of the lateral malleolus, and they are present in about one percent of the general population [11,16,17,18]. SOs have been associated with chronic ankle pain and lateral ankle instability requiring surgery to eliminate the patient’s symptoms [1,15,19,20,21]. In some of these patients, the ATFL attaches directly to the SOs rather than to the fibula, contributing to the instability [22,23]. In clinical decision-making for these patients with chronic pain and lateral ankle instability, clinicians can utilize information from the physical examination, from the plain radiographs, and from advanced imaging. 

MRI, as an advanced imaging modality, has been shown to be useful when evaluating children’s ankle injuries [24,25,26,27,28,29]. MRI can detect occult distal fibula avulsion fractures when plain radiographs are negative. MRI can demonstrate soft tissue edema surrounding symptomatic SOs in adults [6].

For this study, we considered the MRI to be positive if fluid-sensitive sequences showed a line of high intensity between the SO and the tip of the fibula, and the MRI to be negative if the line was of low intensity. We found that 75% of children with positive MRI findings have undergone an operation prior to being released to return to full activity. However, none of those with negative MRI findings have required an operation.

The SOs in our Group 1 patients who underwent surgery were significantly larger than those in Group 2, suggesting that the size of the SOs may contribute to the symptoms of pain and lateral ankle instability. Other investigators have confirmed by surgical observations that the ATFLs are attached directly to the SOs in symptomatic children [7,10,12,22,29]. We found that the MRI sequences in 14 of our 16 Group 1 children showed direct attachment of the ATFLs to the SOs, and our surgical observations confirmed those findings as in case presentations 1 and 3. 

For the patients undergoing an operation, we observed motion between the SOs with the attached ATFL and the fibula. Like our results, other investigators have reported that removal of the SOs coupled with lateral ankle ligament reconstruction eliminated the symptoms and permitted a return to full activity [14,30]. The new information presented in this study is that MRI can provide valuable information concerning the likelihood of which symptomatic children will respond to nonoperative management and which children, most likely, will need to undergo an operation.

We recognize that this study has limitations besides the retrospective collection of data and the relatively small number of patients. We did not identify all children with pain and ankle instability seen at our institution during the study interval. Bias can be introduced with vague terms like chronic ankle pain and ankle instability. Furthermore, the physical therapy we prescribed was not standardized, and we were not blinded to the results of the MRI when discussing treatment options with the family, and that could have biased our presentation of the treatment options to the family. Nonetheless, all families were informed of the treatment options. The families of four patients with positive MRIs chose non-operative treatment. 

## Conclusions

With the available data, our results support the hypothesis that MRI has utility in clinical decision-making for children with recurrent ankle sprains, chronic ankle pain, and radiographic evidence of an SO. Most patients with positive MRI sequences most likely will need to have an operation for excision of the SO and lateral ligament reconstruction prior to being released to full activity. Most patients with negative MRI sequences can be expected to respond to nonoperative treatment such as a structured exercise program focusing on ankle muscle strengthening and proprioception exercises. Further research will help to confirm the results of our initial study.
